# New-Onset Focal Task Specific Oromandibular Dystonia in Association with Quran Recitation: A Case Series

**DOI:** 10.5334/tohm.849

**Published:** 2024-01-11

**Authors:** Jasem Youssef Al-Hashel, Doaa Youssry Soliman, Ismail Ibrahim Ismail

**Affiliations:** 1 Department of Neurology, Ibn Sina Hospital, Kuwait; 2 Department of Medicine, Faculty of Medicine, Health Sciences Centre, Kuwait University, Kuwait

**Keywords:** Focal task-specific dystonia, oromandibular dystonia, Quran

## Abstract

**Background:**

Focal task-specific dystonia is a form of isolated focal dystonia that occurs during the performance of a specific skilled motor task. The occurrence of oromandibular dystonia (OMD) specifically in association with the recitation of Quranic verses have been rarely reported in the literature, in non-native Arabic-speaking patients. This case series describe a rare type of focal task-specific dystonia that occurs exclusively by reciting Quran in native Arabic-speaking patients, which has never been reported, to the best of our knowledge.

**Methods:**

In this case series, we identified five patients with new-onset OMD that was exclusively induced by reciting Quran. Cases were evaluated in our Movement Disorders outpatient clinic at Ibn Sina hospital; the main tertiary neurology center in Kuwait, between 2015 and 2023.

**Results:**

Five cases (3 males, 2 females) were identified in this study. Mean age of onset of the symptoms was 52.3 ± 4.1 years, while the median duration of the symptoms prior to diagnosis was 3 years. All patients were native Arab-speaking, with no previous history of other types of dystonia. No identifiable risk factors could be obtained including exposure to dopamine blocking agents or antipsychotics, or history of oral or dental surgery. Patients underwent a full clinical, laboratory, and radiological evaluation. All patients had OMD dystonia in varying forms and severity, while two patients had additional spasmodic dysphonia/ blepharospasm on progressive recitation. Most patients had minimal improvement with combination of oral medications and speech therapy. Four patients received botulinum toxin injections with better results.

**Discussion:**

The mental and physical stress in attempting to recite the Quranic verses could have contributed to the development of OMD. Moreover, the increased demand on the muscles of the jaw, lips, and tongue during recitation can trigger the dystonic symptoms.

**Highlights:**

OMD exclusively during Quran recitation is a rare phenomenon, and expands the spectrum of task-specific focal dystonia described in the literature. It was found to be distressing to the patients and a challenge to treat. Prompt recognition could minimize unnecessary testing and procedures, and facilitate earlier treatment.

## Introduction

Dystonia is a movement disorder that is characterized by disabling sustained or intermittent, involuntary muscle contractions, resulting in abnormal repetitive, twisting movements, abnormal postures, or both. It can affect a specific body region (focal dystonia), multiple body regions (segmental, multifocal, hemibody or generalized dystonia), or during the performance of a specific activity (task-specific dystonia) [1].

Focal task-specific dystonia (FTSD) is an interesting type of isolated focal dystonia that is triggered, at least initially, by the performance of a specific skilled motor task. It has been described in musicians, writers, computer keyboard users, and athletes (e.g. table tennis players, golfers) [2]. Oromandibular dystonia (OMD) is a type of focal dystonia that involves the masticatory or lingual muscles, and is subdivided into jaw closing dystonia, jaw opening dystonia, jaw deviation dystonia, jaw protrusion dystonia, lingual dystonia, or a combination of these subtypes [3].

In this study, we describe five cases of new-onset task specific OMD in association with the recitation of the holy Quran (the central religious text of Islam, and believed by Muslims to be a revelation from God). This phenomenon has been rarely reported in the literature, and mainly in few case reports of non-native Arabic-speaking patients. We aim to highlight the importance of this unique phenomenon, to facilitate earlier diagnosis and treatment.

## Methods

In this case series, five patients with new-onset OMD that was exclusively induced by reciting Quran were identified. Cases were evaluated in our Movement Disorders outpatient clinic at Ibn Sina hospital; the main tertiary neurology center in Kuwait, in the period between January 1, 2015 and October 1, 2023. Patients were identified from the assigned period, and their medical records were retrieved. Demographic data included age, gender, and nationality. Data regarding dystonia onset, type, associated other types of dystonia, presence of sensory tricks, treatment, and response to treatment, were collected. Their detailed history, neurological examination, laboratory investigations, and brain imaging were evaluated.

Study protocol and informed consent were reviewed and approved by the Scientific Research Committee of Department of Neurology, Ibn Sina hospital, Kuwait. Written Informed consent was obtained from each patient.

## Statistical Analysis

All data were analyzed using MedCalc® Statistical Software version 20.111 (MedCalc® Software Ltd, Ostend, Belgium; https://www.medcalc.org; 2022). Descriptive statistics are reported as numbers, mean ± standard deviation, and median.

## Results

Five patients (3 males, 2 females) were identified in our study. The mean age of onset of the symptoms was 52.3 ± 4.1 years, while the median duration of the symptoms prior to diagnosis was 3 years. All patients were native Arab-speaking (5 Kuwaiti, 1 Egyptian), with no previous history or family history of other types of dystonia. All patients had OMD dystonia in varying forms and severity that was triggered exclusively by reciting the Quran. They did not show any dystonic movements while speaking normally, eating or singing.

No identifiable risk factors could be obtained including exposure to drugs such as dopamine blocking agents or antipsychotics, trauma, or history of oral or dental surgery. All patients underwent a full clinical, laboratory, and radiological evaluation which were all normal.

The main type was jaw closing dystonia, which was prevailing in all cases. A combination of jaw closing, jaw opening, and jaw deviation dystonias were seen in two patients. One patient had additional lingual dystonia. Patients reported no chewing difficulties, dysphagia, breathing difficulties, or alteration in vocalization other than while reciting the Quran. Two patients had additional spasmodic dysphonia/ blepharospasm on progressive recitation. OMD in those patients were not painful, and did not occur during normal speech. Two patients had sensory tricks (geste antagoniste): one patient with inserting the temple tips of his eyeglasses inside his mouth during recitation, while the other touches his cheeks or chin with his finger.

Most patients (n = 4) had minimal to no improvement with a combination of oral medications (clonazepam, trihexylphenidyl, baclofen, levodopa) and speech therapy. Four patients received Onabotulinumtoxin A (Botox) injections, with good to partial improvement of their symptoms.

The demographic and clinical characteristics of our cohort are summarized in Table 1.

**Table 1:** Clinical and demographic characteristics of patient population (n = 5).


PATIENTS	AGE (YEARS)	GENDER	DISEASE DURATION (MONTHS)	OMD TYPE	GESTE ANTAGONISTE	OTHER TYPES OF DYSTONIA	MEDICAL TREATMENT	TYPES OF MEDICAL TREATMENT	RESPONSE TO MEDICAL TREATMENT	BOTULINUM TOXIN INJECTION	RESPONSE TO BOTULINUM TOXIN

**1**	42	F	12	Jaw closing	Y	N	Y	Clonazepam, baclofen and trihexyphenidyl	Minimal improvement	Y	Good improvement

**2**	54	M	18	Jaw closing	N	N	Y	Levodopa, clonazepam, baclofen, trihexyphenidyl	No improvement	Y	Partial improvement

**3**	38	F	9	Jaw closing, Jaw opening	N	Spasmodic dysphonia	Y	Clonazepam and baclofen	Minimal improvement	Y	Partial improvement

**4**	49	M	36	Jaw closing, Jaw deviation	N	Blepharospasm	Y	Clonazepam, baclofen and trihexyphenidyl	Minimal improvement	Y	Good improvement

**5**	58	M	48	Jaw closing, Lingual	Y	N	Y	Clonazepam, baclofen and trihexyphenidyl	No improvement	N	N/A


## Case 1

Mrs. S is a 42-year-old Kuwaiti female, with no relevant past medical history, presented with isolated jaw closing OMD of 1-year duration, without any concomitant neurological signs or abnormalities. Symptoms were exclusively triggered by reciting the Quranic verses, and were absent during regular speech, singing, or eating. The severity increased while attempting to recite faster or louder during prayers. She underwent medical treatment with a combination of clonazepam (4 mg/day), baclofen (30 mg/day) and trihexyphenidyl (4 mg/day), over a 3 months period. She had minimal improvement of her symptoms and medications had to be tapered. Onabotulinumtoxin A was injected (10 units in both masseter muscles, and 25 units in both temporalis muscles), and she showed good improvement of her symptoms. Her OMD before and after treatment can be seen in Video 1.

**Video 1:** The video shows a 42-year-old female, with jaw closing dystonia triggered by recitation of Quranic verses, before and after Onabotulinumtoxin A (Botox) injection. The second half of the video illustrates significant improvement of her symptoms during recitation.

## Discussion

In this case series, we report an uncommon phenomenon of OMD occurring exclusively during Quran recitation in five native Arabic-speaking patients, further expanding the spectrum of FTSD. This unique phenomenon showed only mild improvement with medical treatment and speech therapy, however, Onabotulinumtoxin A had an added beneficial effect on the symptoms. FTSD in the orofacial region has been commonly reported with certain occupations such as professional musicians (e.ge. embouchure dystonia, opera singers), auctioneers, telemarketers, and bingo callers [3,4]. Non-occupational task-specific dystonia is more uncommon, and have been reported with certain actions such as mastication, and speech-related dystonia [5,6].

OMD with Quran recitation had been rarely published in the literature, and no previous cases were reported in native Arabic-speaking patients, to the best of our knowledge. Ilic and colleagues [7] had reported a case of a 47-year-old man, living in Germany but of Turkish descent who was fluent in both languages, who developed OMD only during Islamic prayers in Arabic language.

Kutty and colleagues [8], described the same clinical presentation in six non-native Arabic-speaking patients who developed OMD while reciting Quran, during and outside Islamic prayers. The authors attributed the findings to the extra mental and physical effort while attempting to recite the Quranic verses accurately, as none of the patients were native Arabic-speaking. Similarly, Bonanni and colleagues [9] reported a 42-year-old man with lower lip dystonia while performing Buddhist mantras.

Quranic recitation often follows specific rules of proper pronunciation, intonation, and rhythm, and could be perceived as stressful even for native Arabic-speaking individuals. This unique style of recitation, known as Tajweed, gives the Quranic verses a distinct and melodious quality. This rhythmic recitation is different from everyday conversational speech, as it imposes higher attention to details, as even the slightest mispronunciation can alter the meaning of a verse.

Moreover, many Muslims commit the Quran to memory, and aim at memorizing it word-for-word, as a significant life achievement, which requires intense practice schedules, and carries a high level of self-imposed pressure and devotion to recite it accurately. This pressure or stress is different from normal speech, which is a more relaxed and spontaneous form of communication. Furthermore, the increased demand on the muscles of the jaw, lips, and tongue during recitation can trigger the dystonic symptoms.

The exact pathophysiology is still unclear, although recent evidence suggests that both genetic and environmental factors are important. Numerous studies have indicated some abnormalities of the basal ganglia or its connections, loss of cortical inhibition at various sensorimotor levels, abnormal plasticity regulation, and impaired sensorimotor processing [10,11].

The basal ganglia and its interconnected structures play a crucial role in motor control and movement initiation. In FTSD, studies have shown abnormalities in the basal ganglia, particularly the putamen and caudate nucleus, have been observed. These abnormalities might involve dopamine dysfunction, changes in neuronal activity, or altered gating of sensory information [11,12].

Moreover, cortical abnormalities of the primary motor cortex and supplementary motor area (SMA) have shown reduced activity during the targeted task in FTSD patients, suggesting a possible failure of inhibition of the unwanted motor commands, as these areas are responsible for planning and executing movements. Additionally, abnormal sensory representation of the affected body part has been observed in the somatosensory cortex [13].

Finally, abnormal plasticity has been suggested in different studies to play a key role in FTSD. This abnormal plasticity in the sensorimotor pathways can lead to excessive strengthening of some neural connections and weakening of others, resulting in involuntary activation or inhibition of these unwanted pathways when attempting the task, thus contributing to the dystonic movements during the specific task [14].

This study had some limitations that need to be highlighted. First, we are aware that our case series had a small number of patients, however, and given the rarity of the reported finding, we believe that reporting such a phenomenon could facilitate its early recognition and diagnosis, and hence, its early treatment. Second, we relied on patients’ subjective reporting of the response to treatment for the evaluation of the level of improvement and impairment. A reliable validated scale could have confirmed our findings in this point. Finally, data regarding the frequency and duration of Quran recitation by the patients before developing dystonia was not available, thus a conclusion could not be drawn between them. It is hard to accurately estimate the frequency and duration of Quran recitation, as most Muslims begin to study, learn, and memorize Quran at a very young age, and continue to do so on variable paces as they grow up.

In conclusion, OMD exclusively during Quran recitation is a rare phenomenon that expands the spectrum of focal task-specific dystonia described in the literature. The mental and physical stress in attempting to recite the Quranic verses, in addition to the increased demand on the muscles of the jaw, lips, and tongue during recitation, could have triggered the dystonic symptoms. This new phenomenon was found to be distressing to the patients and a challenge to treat. Prompt recognition could facilitate earlier treatment and improving patients’ quality of life.
